# Low-intensity exercise combined with sodium valproate attenuates kainic acid-induced seizures and associated co-morbidities by inhibiting NF-κB signaling in mice

**DOI:** 10.3389/fneur.2022.993405

**Published:** 2022-09-21

**Authors:** Yuxiang Jia, Lele Tang, Yu Yao, Limin Zhuo, Dongxiao Qu, Xingxing Chen, Yonghua Ji, Jie Tao, Yudan Zhu

**Affiliations:** ^1^School of Medicine, Shanghai University, Shanghai, China; ^2^Department of Neurology and Neurosurgery, Putuo Hospital, Shanghai University of Traditional Chinese Medicine, Shanghai, China

**Keywords:** seizures, low-intensity exercise, sodium valproate, co-morbidities, TLR4/NF-κB pathway, inflammatory factors

## Abstract

Sodium valproate (VPA) is a broad-spectrum anticonvulsant that is effective both in adults and children suffering from epilepsy, but it causes psychiatric and behavioral side effects in patients with epilepsy. In addition, 30% of patients with epilepsy develop resistance to VPA. At present, regular physical exercise has shown many benefits and has become an effective complementary therapy for various brain diseases, including epilepsy. Therefore, we wondered whether VPA combined with exercise would be more effective in the treatment of seizures and associated co-morbidities. Here, we used a mouse model with kainic acid (KA)-induced epilepsy to compare the seizure status and the levels of related co-morbidities, such as cognition, depression, anxiety, and movement disorders, in each group using animal behavioral experiment and local field potential recordings. Subsequently, we investigated the mechanism behind this phenomenon by immunological means. Our results showed that low-intensity exercise combined with VPA reduced seizures and associated co-morbidities. This phenomenon seems to be related to the Toll-like receptor 4, activation of the nuclear factor kappa B (NF-κB), and release of interleukin 1β (IL-1β), tumor necrosis factor α (TNF-α), and IL-6. In brief, low-intensity exercise combined with VPA enhanced the downregulation of NF-κB-related inflammatory response, thereby alleviating the seizures, and associated co-morbidities.

## Introduction

Epilepsy is a brain disorder that affects over 70 million people worldwide. This disorder is characterized by the abnormal discharge of neurons (1, 2). Currently, antiepileptic drugs, such as sodium valproate (VPA), are the most common form of treatment. After more than a century from its discovery, as a result of its anticonvulsant effects, VPA is still a drug of choice for epilepsy in children and adults with either general or focal seizures (3). However, antiepileptic drugs cause psychiatric and behavioral side effects (4, 5) and drug resistance in ~ 30% of patients with epilepsy (6). Therefore, it is urgent to find a non-pharmacological intervention that can reduce the development rate of drug-resistant epilepsy and the side effects associated with antiepileptic drugs. Noteworthily, physical exercise is a complementary therapy with beneficial effects for epilepsy (7–9). The beneficial effects of low-intensity physical exercise on the brain have been demonstrated in mouse models of disorders like Alzheimer's disease (AD), stroke, and depression (10–13). Clinical studies have found that more than half of the patients never experience a seizure during or after physical exercise, including 10% of patients who have frequent seizures (14); therefore, physical exercise seems to reduce the severity of seizures. Therefore, we evaluated whether low-intensity physical exercise combined with VPA (LE-VPA) would be more effective for treating seizures and other epilepsy-associated co-morbidities in mice.

Epilepsy pathogenesis is complex, but evidence suggests an increased number of microglial cells in the Cornu Ammonis 1 (CA1) and Cornu Ammonis 3 (CA3) layers of the hippocampus in patients with epilepsy (15–17). Therefore, microglial-mediated neuroinflammation may be involved in its pathogenesis (18, 19). Microglial cells are brain-resident immune cells that regulate mechanisms essential for cognitive functions. A variety of receptors are distributed on the surface or in the cytoplasm of microglial cells (20, 21), including Toll-like receptor 4 (TLR4). TLR4 belongs to one of the subfamilies of pattern recognition receptors that recognize invading pathogens and endogenous harmful stimuli, thus, activating the transcription factor nuclear factor κB (NF)-κB pathway (22), and releasing large amounts of inflammatory proteins (e.g., interleukin 1-β [IL-1β], interleukin 6 [IL-6], tumor necrosis factor α [TNF-α], and chemokines) (23–25). VPA induces epigenetic signaling that involves NF-κB-related inflammatory responses in the frontal cortex and hippocampus (26, 27). Meanwhile, it has been reported that physical exercise alleviates inflammatory lung injury (28) and osteoarthritis (29) by inhibiting NF-κB signaling. Therefore, we studied whether LE-VPA alleviates seizures and associated co-morbidities by inhibiting TLR4/NF-κB-related inflammatory responses.

In this study, we aimed to examine whether LE-VPA exerted an ameliorative effect in a kainic acid (KA) mouse model for seizures and associated co-morbidities (i.e., cognitive dysfunction, motor impairment, depression, and anxiety). In addition, we hypothesized that the positive effects of LE-VPA were associated with the inhibition of TLR4/ NF-κB and lower expression levels of IL-1β, TNF-α, and IL-6.

## Materials and methods

### Animals

Eighty male, 2-month-old C57BL/6 mice (Jiesijie Laboratory Animal, Shanghai, China), weighing 20–25 g, were used. Except for a control group of eight mice, epilepsy was induced in the remaining mice by intraperitoneal injection of kainic acid (KA; 30 mg/kg) according to body weight. The symptoms of injected mice were judged according to the Racine scale, and the mice that were successfully modeled were divided into four groups (30), with eight mice in each group: model group, drug group, runner group, and running combined with drug treatment group (combination group). In our experiment, out of the 60 mice that received KA injection, 32 survived, with a death rate of around 50% due to KA. All animal experiments were performed according to the National Institutes of Health (NIH) Guide for the Care and Use of Laboratory Animals (NIH Publication No. 80–23, revised 1996) and were approved by the Institutional Animal Care and Use Committee of Shanghai University of Traditional Chinese Medicine.

### Treatment program

In this study, the mice performed low-intensity physical exercise on treadmills (SANS, SA 101). It has been proven that the average maximal speed of young mice is 70 cm/s (42 m/min), and low-intensity exercise is defined as around 35–40% (14.7 m/min−16.8 m/min) of the maximal capacity (31). In addition, we observed that the mice ran at 15 m/min without any discomfort. Therefore, the maximal intensity of the mouse treadmill was set to 15 m/min. Runners were assigned to run 5 m/min for 10 min on the first day, and the running speed was gradually increased to 15 m/min for 20 min from second to seventh day and 15 m/min for 60 min from eighth to 28th day. The treadmill exercise was performed 5 days per week.

The drug group and the combination group received an intraperitoneal injection of VPA (Sigma, St Louis, MO, USA) dissolved in 0.9% NaCl, at 30 mg/kg per day, for 28 days. The model group were injected with the same volume of normal saline daily for 4 weeks (Table 1).

**Table 1:** Exercise and treatment program.

**Group**	**Pretreatment**	**Exercise**	**VPA (ip)**	**Saline (ip)**	**Duration (day)**
Control	-	-	-	+	28
Model group	KA, ip	-	-	+	28
Drug group	KA, ip	-	+	-	28
Runner group	KA, ip	+	-	-	28
Combination group	KA, ip	+	+	-	28

### Video monitoring

Cameras were used to monitor the mice moving freely in an observation bucket. Each mouse was placed in a transparent observation bucket equipped with a surveillance camera (Shanghai JiLian Web Tech Ltd., Shanghai, China) for 9 h for continuous observation. Using the Racine scale, the duration and frequency of epileptic seizures in each mouse during this period were analyzed and recorded.

### Morris water maze

The Morris water maze (MWM) (32) is a circular pool with a diameter of 120 cm and a height of 60 cm, a water level of 35 cm, and a temperature of 22–24 °C. The mice were trained on the MWM with one trial per day for 6 days. A platform was hidden 1 cm below the surface of the water, which was made opaque with white non-toxic paint. Each trial lasted until the mouse found the platform or for a maximum of 60 s. At the end of each trial, mice were allowed to rest on the platform for 60 s. The time to reach the platform (latency), length of the swim path, and swim speed were recorded semi-automatically using a video tracking system (Shanghai JiLian Web Tech Ltd.).

### Y maze

The Y maze (Shanghai JiLian Web Tech Ltd.) has three arms in total, with a 120° angle between the two arms (33). An infrared camera tracking system was installed above the maze to record the process, using supporting software. In the experiment, the mice were placed toward the center of the Y maze from the end of one arm and were allowed to explore freely for 10 min. In the spontaneous alternation reaction, the complete entry of the body of the mouse into the arm was recorded as a standard entry, and the continuous entry of the animal into the three different arms was defined as successful exploration.

Alternation rate (%) = correct entry time / (total entry times-2) × 100.

### Open-field testing

The open-field test is used to measure anxiety-like behavior in animals. This test used a camera to measure the movement of the test animal in the peripheral and central zones (20 × 20 × 20 cm) of a 42 × 42 × 42 cm polyvinyl chloride box for 10 min. A video tracking program (Stoelting Co., Wood Dale, IL, USA) was used to record and measure the total distance traveled, the time spent in the center of the open-field arena, and the distance moved in the center of the open-field arena in each trial. The time spent in the center and distance traveled in the center of the arena were used as measures of anxiety-like behavior (34, 35).

### Catwalk gait analysis

The Catwalk System (Catwalk XT, Noldus Information Technology, Wageningen, Netherlands) consists of an enclosed walkway (glass plate) illuminated by fluorescent light. The system was equipped with a high-speed color camera connected to a computer using appropriate detection software (CatwalkXT9.1). Animals were individually placed on the walkway, and each animal was allowed to move freely in both directions. To detect all parameters used in the experiments, the camera gain was set to 20, and the detection threshold was set to 0.1. All runs with a run duration between 0.50 and 5.00 s for a complete walkway and a maximum allowed speed variation of 60% were considered as successful runs. For each animal, three compliant runs were performed per trial. Compliant runs were classified for all limbs and were statistically analyzed. The software can detect several dynamic parameters of an animal's walk (36): we focused on swing speed (average stride time), body speed (distance traveled/time), print area (measurements of complete paw prints), and mean intensity (average pressure applied by single-paw contact with the floor).

### Local field potential

Mice were deeply anesthetized and placed in a stereotaxic frame (Narishige, Tokyo, Japan). Multi-channel electrodes were inserted into the hippocampus of mice based on a mouse brain map (anteroposterior: −2.0 mm posterior to bregma, ventral: 1.5 mm ventral to the dura surface, and lateral: 1.5 mm lateral to the skull midline). After the mice woke up from anesthesia and moved freely, the local field potential (LFP) recordings were obtained. Data were collected using an *in vivo* multi-channel recording system, amplified 1,000 times by a preamplifier, with a wave amplitude range of −2 to +2V and a filtering range of 1.6–100 Hz. The LFP recording frequency was 1,000 Hz, and the recording time was not < 30 min. LFP was exported in Pl2 format, and the Offline Sorter V4 software was used for visual previews. The LFP analysis selected the same channel using MATLAB (MathWorks, Natick, MO, USA) to export data. Five different frequencies of circadian rhythms were decomposed: δ (0.4–4 Hz), θ (4–8 Hz), α (8–15 Hz), β (15–30 Hz), and γ (> 30 Hz). The Welch Hamming window and fast Fourier transform methods were used to calculate the frequency domain information of the LFP. The power spectral density (PSD) was calculated as follows:


(1) $$\begin{array}{l} \int_{-\infty }^{+\infty }x^{2}\left(t\right)dt=\frac{1}{2\pi }\int_{-\infty }^{+\infty }|X\left(jw\right){|}^{2}d\omega \end{array}$$

(2) $$P=\frac{1}{2T}\int_{-T}^{T}x^{2}\left(t\right)dt=\frac{1}{2\pi }\int_{-\infty }^{+\infty }\frac{1}{2T}{\left|X_{T}\left(\omega \right)\right|}^{2}d\omega$$

### Immunofluorescence and immunohistochemistry

After acute anesthesia with chloral hydrate, mice were transcardially perfused with ice-cold 4% paraformaldehyde (PFA). The dissected brains were post-fixed overnight in 4% PFA and were then transferred to 30% sucrose buffer for 48 h. Subsequently, the brains were frozen in Tissue-Tek OCT compound (Sakura Finetek, Torrance, CA, USA) and sliced at 20 μm using a cryostat (Leica Biosystems, Nußloch, Germany). The sections were washed with phosphate-buffered saline (PBS, pH 7.4), incubated with 0.3% Triton in PBS at room temperature for 0.5 h, and incubated with blocking solution (Beyotime Biotech, Shanghai, China) at room temperature for 1 h. Then, the sections were treated with appropriate primary antibodies at a suitable concentration in blocking solution at 4°C for overnight. After washing with PBS three times, the sections were incubated with secondary antibody at 37°C for 1 h. After washing again with PBS three times, the sections were incubated with DAPI (Beyotime, 1:1) at room temperature for 10 min. The sections were then washed, mounted, and covered with a lid. The images were analyzed using Image-Pro Plus 6.0 software (Media Cybernetics).

The steps of immunohistochemical procedure on the first day of experiment were equivalent to those of immunofluorescence. On the next day, the steps of immunohistochemical procedure were different. First, brain slices were incubated with secondary antibodies and stained with 3, 3'-diaminobenzidine (DAB) using the SABC and DAB staining kit (Wellbio, Shanghai, China), according to the instructions. Then, hematoxylin was used for counterstaining. The differentiation solution was terminated. Finally, dehydration, transparency, sealing, and observation were performed. A light microscope (ECLIPSE E600; Nikon, Tokyo, Japan) was used to observe the sections, and at least five panoramic images of the hippocampus were taken from each group. Image-pro Plus 6.0 software was used to analyze the intensity of staining areas in each group (Media Cybernetics, MD, USA).

The primary antibodies used in this study were rabbit anti-IL-1β antibody (Abcam, Cambridge, MA, USA; EPR23851-127; 1:100), rabbit anti-TNF-α (Abcam, EPR20972; 1:100), mouse anti-iba1 (Abcam, EPR16589; 1:100), rabbit anti-IL-6 (Abmart, Shanghai, China; P05231; 1:100), rabbit anti-NLRP3 (Affinity, Shanghai, China; DF7438; 1:100), rabbit anti-TLR4 (Affinity, Shanghai, China; AF7017; 1:100), and rabbit anti-NF-Kb-p65 (Affinity, Shanghai, China; AF5006; 1:100). The secondary antibodies used were Alexa Fluor 488 goat anti-mouse (Abcam, 1:200) and Alexa Fluor 647 goat anti-rabbit (Abcam,1:200).

### Enzyme-linked immunosorbent assay

The mouse brains were dissected, and hippocampal tissues were quickly removed on ice. Blood was washed away with pre-cooled fresh PBS. Hippocampal tissues were homogenized in PBS and centrifuged at 45,000 × *g* for 30 min at 4°C. We used enzyme-linked immunosorbent assay (ELISA) kits to measure cytokines including IL-1β, TNF-α, and IL-6, according to the manufacturer's protocols. The results were obtained using a multifunction microplate meter.

### Statistical analysis

Data were expressed as mean ± standard error of the mean. Statistical differences were analyzed using two-way analysis of variance or three-way analysis of variance (followed by Tukey's multiple comparison test). For multiple comparisons, we selected “simple effect within row“ or ”main column effect,” respectively, depending on the purpose, and compared each column mean with every other column mean. Data were considered significant at *P* < 0.05. Statistical analyses were performed using GraphPad Prism (version 6.07, GraphPad, San Diego, USA), Excel 2016 (Microsoft, WA), and Adobe Photoshop CC 2018 (Adobe Systems, San José, CA, USA).

## Results

### Experimental design

**Figure 1:**
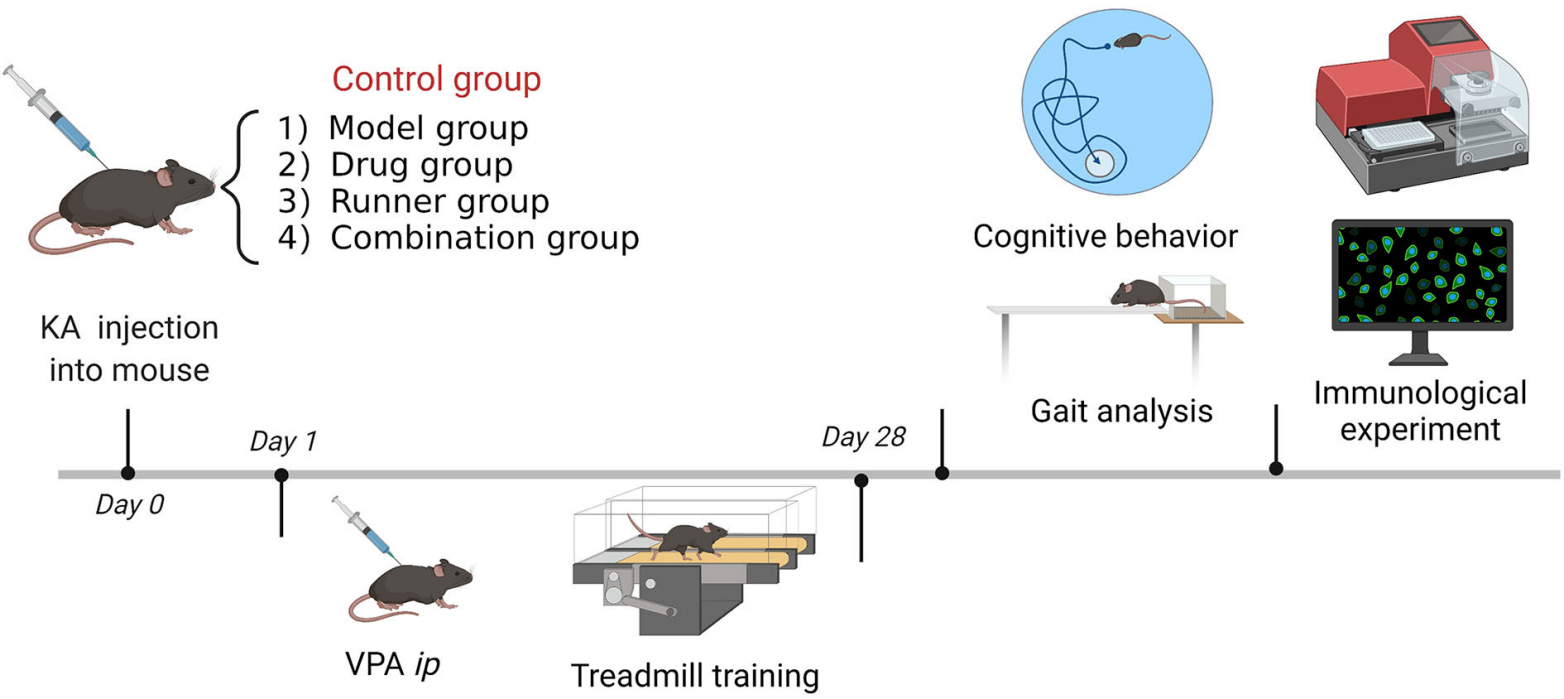
Flowchart of the experiment.

Mice were assigned to five groups (*n* = 8) according to the experimental protocol. The animals were maintained under their respective experimental conditions for 28 days. Mice were tested in behavioral experiments, and LFP recordings were made between days 29 and 37. After sacrificing the mice, we collected brain tissue for immunological experiments (Figure 1).

### LE-VPA provided better control of mild seizures

**Figure 2:**
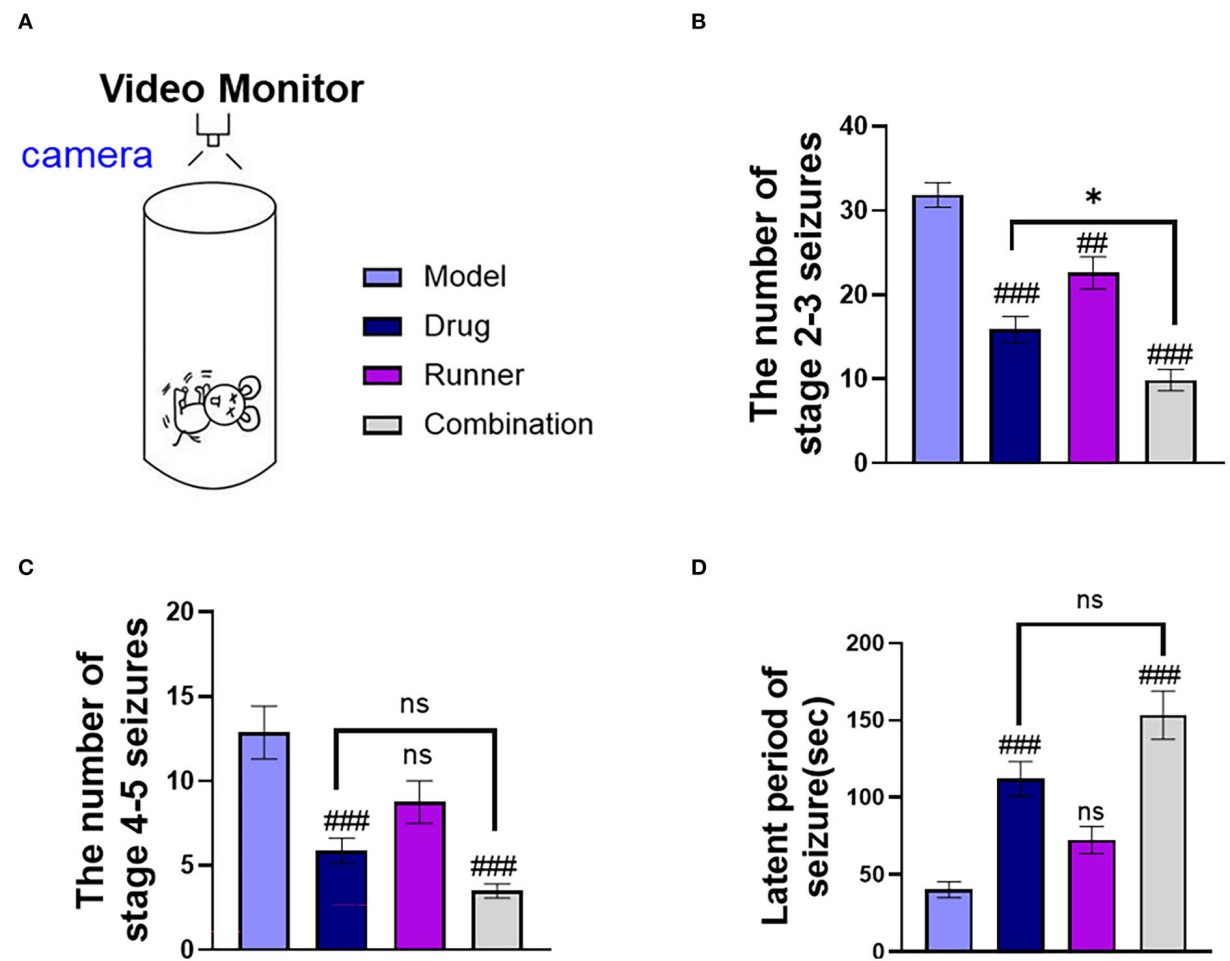
Seizure status of mice in each group. **(A)** A monitoring camera for continuous observation for 9 h. **(B,C)** The number of stages 2–3 and stages 4–5 seizures. **(D)** Latent period of tonic–clonic seizure in each group. Data were shown as mean ± SEM. ns > 0.05, *P < 0.05, ^##^*P* < 0.01, ^###^*P* < 0.001, comparison of the model group with each treated group (two-way ANOVA).

Mice were intraperitoneally injected with KA and presented with status epilepticus (SE) 15–30 min later. After a latency of 14–21 days, mice presented with spontaneous epilepsy (37). We used video cameras to track the number of seizures and the latency of tonic–clonic seizures in each group for the continuous 9-h video monitoring (Figure 2A). In the statistics, the number of mild seizures (stages 2–3) (Figure 2B) in the three treated groups showed different degrees of improvement (drug: *P* < 0.001, runner: *P* < 0.01, combination: *P* < 0.001, *n* = 8), and LE-VPA had greater advantages than VPA alone (*P* < 0.05). The number of severe seizures (stages 4–5) (Figure 2C) showed remission in only two groups that are treated with VPA (drug: *P* < 0.001, combination: *P* < 0.001), and the effect of running appeared to be modest (*P* > 0.05). The statistics of seizure latency (Figure 2D) also showed a significant difference among VPA-treated groups (drug: *P* < 0.001, combination: *P* < 0.001), but there was no significant difference between LE-VPA and VPA alone group (*P* > 0.05). Taken together, LE-VPA therapy significantly reduced the number of mild seizures in KA mice.

### LE-VPA improved epilepsy and associated co-morbidities like cognitive impairment, depression, and anxiety

We also evaluated whether LE-VPA could alleviate the cognitive impairment, depression, and anxiety that are associated with epilepsy.

**Figure 3:**
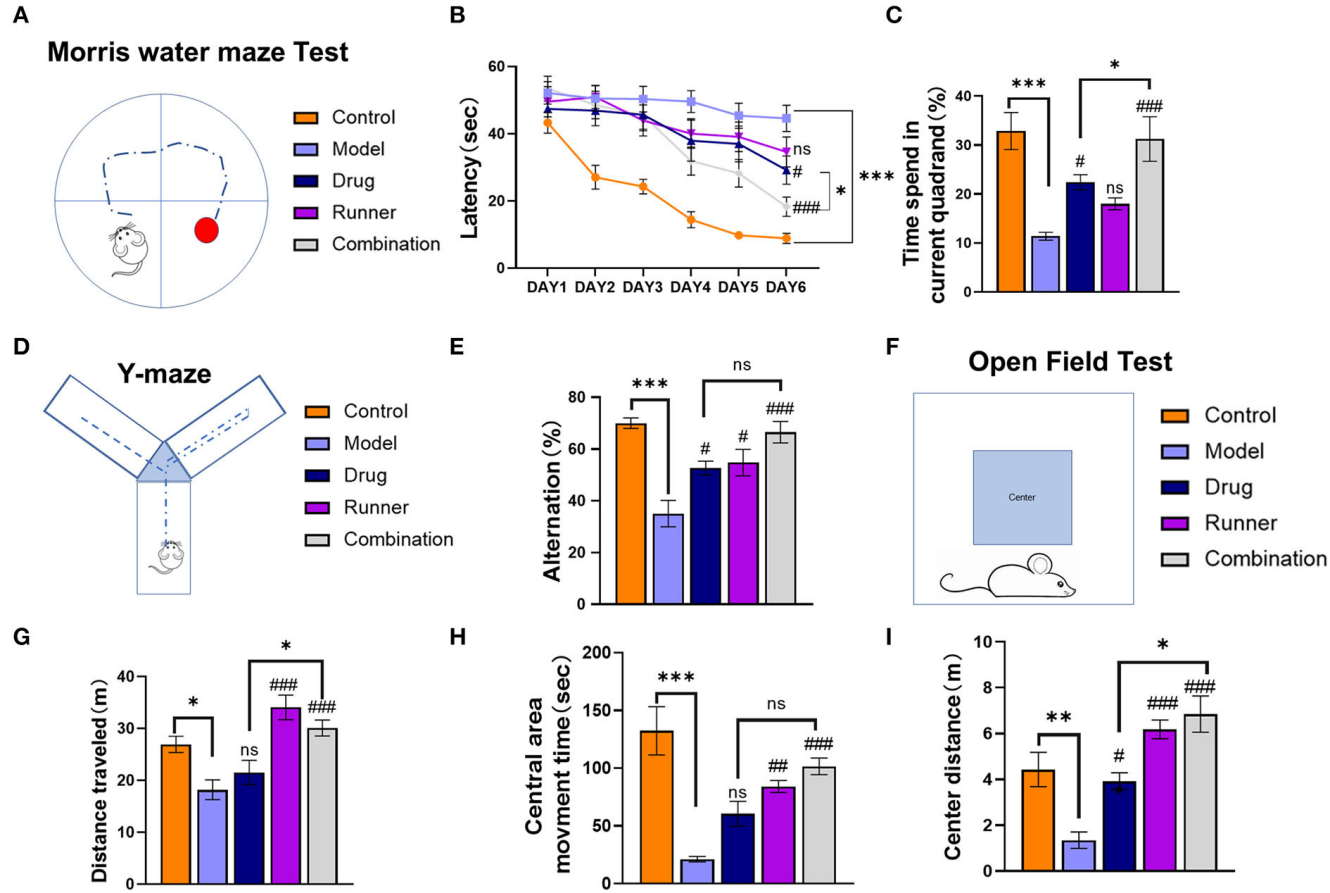
Cognitive impairment, anxiety, and depression in mice of each group. **(A)** Schematic of the Morris water maze task. **(B,C)** The 6-day latency of each group and time spent in the current quadrant on day 6. **(D)** Schematic of the Y-maze test task. **(E)** The spontaneous alternation in behavior of each group. **(F–I)** The open-field test was performed to record the total distance traveled throughout the open-field arena, entries into the center zone, duration spent within the center zone, and distance traveled within the center zone. Data were shown as mean ± SEM. ns > 0.05, *P < 0.05, **P < 0.01, ***P < 0.001; ^#^*P* < 0.05, ^##^*P* < 0.01, ^###^*P* < 0.001, comparison of the model group with each treated group (B: three-way ANOVA or two-way ANOVA was used for the rest of the data).

Mice in each group received the corresponding treatment for 28 days, followed by MWM that was conducted for 6 days (Figure 3A). During the first 5 days of the training period, the software recorded the latency for each mouse which provided evidence for spatial learning. The 6th day was the test period, and the time to find the platform for the first time was recorded as the latency on day 6. The time spent in the correct quadrant represented memory retention. The latency of healthy mice shortened over training, indicating that mice had a normal spatial learning ability, while the latency of mice in the KA model group remained unchanged or was not significantly shortened, indicating that epileptic mice had a spatial learning ability disorder (Figure 3B). After 5 days of training, the experimental performance of each group of mice on the last day was as follows: The time to find the platform was approximately 20 s shorter in the drug group than in the model group (*P* < 0.05, *n* = 6), while that in the LE-VPA group was about 30 s shorter (*P* < 0.001, *n* = 6). The difference between the drug and LE-VPA groups was also significant (*P* < 0.05). We then compared the duration that each group spent in the correct quadrant on day 6 (Figure 3C). The model mice spent a shorter time in the quadrant as compared to control mice, indicating memory dysfunction (*P* < 0.001, *n* = 6). This could be improved by 5 % after drug therapy alone (*P* < 0.05, *n* = 6). The improvement effect of the LE-VPA therapy was more obvious: The time was increased by 20% (*P* < 0.001). The effect of LE-VPA also differed significantly from that of VPA alone in this respect (*P* < 0.05).

In the Y-maze task (Figure 3D), based on the mouse's movements, the software automatically calculated the rate of alternation for each group, which represents the mouse's working memory (Figure 3E). Compared to control mice, the spontaneous alternation rate was lower in the model group (*P* < 0.001, *n* = 6). With drug alone and running alone therapy, mice showed a 10% (both *P* < 0.05, *n* = 6) increase in spontaneous alternation rates, respectively, whereas combination group seemed to have a better effect (*P* < 0.001). In addition, there was no significant difference between LE-VPA and VPA alone (*P* > 0.05).

Next, the mice were subjected to an open-field test (Figure 3F). Compared with control mice, KA mice moved significantly less distance (*P* < 0.05, *n* = 8) and had depression-like behavior (Figure 3G). After different ways of treatment, this condition has been alleviated in runner and combination groups (both *P* < 0.001, *n* = 8), showing LE-VPA is more effective than VPA therapy alone (*P* < 0.05, *n* = 8). We counted the movement time and distance of each group in the central area. We found that the movement time (*P* < 0.00) and distance (*P* < 0.01) in the center of the model group were significantly shorter than healthy mice, indicating that epilepsy could cause anxiety-like behavior in mice (Figures 3H,I). After different treatments, the time (runner: *P* < 0.01, combination: *P* < 0.001, *n* =7) and distance (drug: *P* < 0.05, runner: *P* < 0.001, combination: *P* < 0.001, *n* =7) of movement in the center of each group increased significantly. There were significant differences between the LE-VPA group and VPA group in center distance (*P* < 0.05).

In conclusion, compared with drug alone treatment, LE-VPA was more effective in improving the learning ability and memory retention as well as ameliorating epileptic-induced depression and anxiety-like behavior in KA mice.

### Dyskinesia of epilepsy was ameliorated by LE-VPA

**Figure 4:**
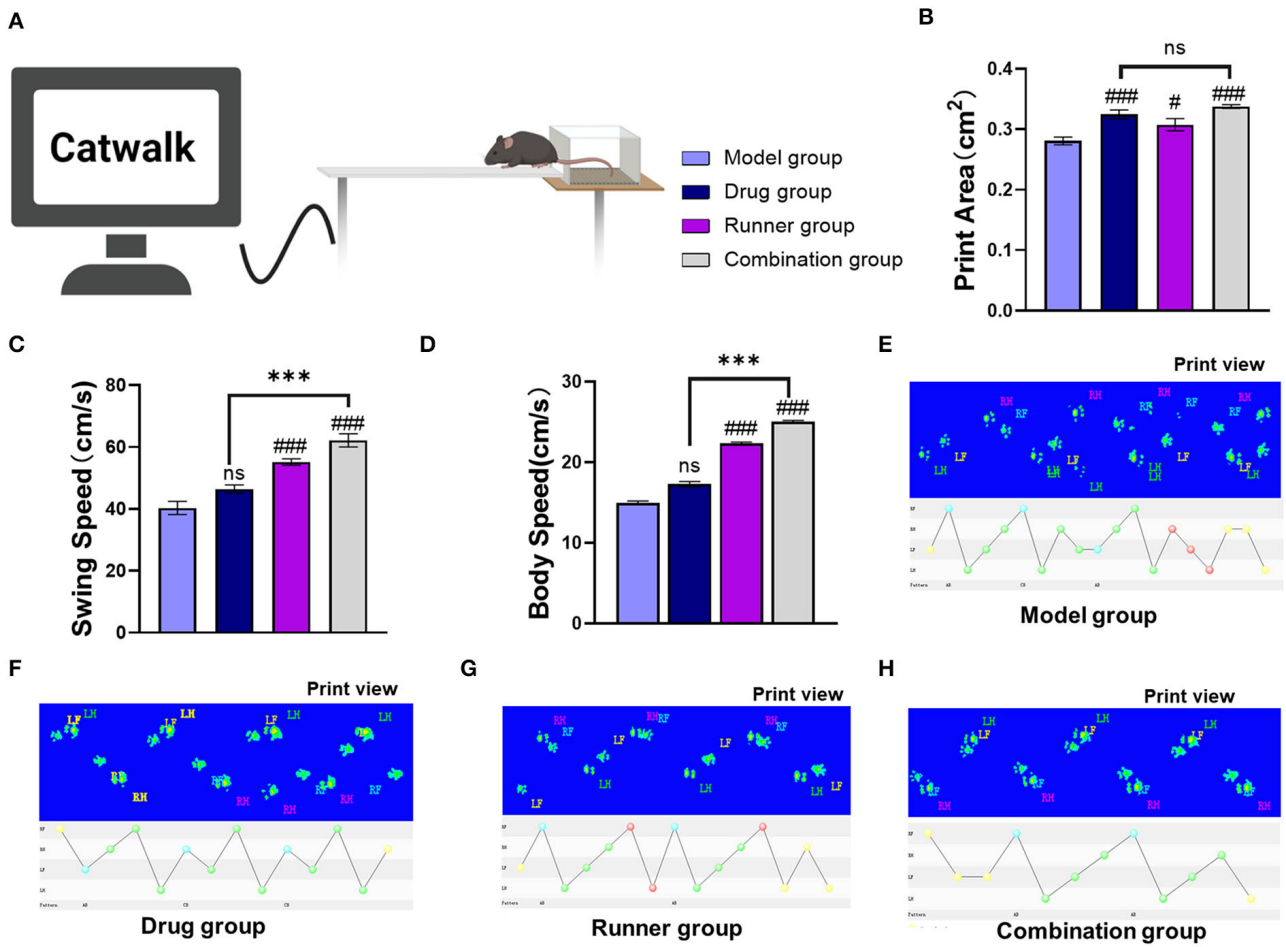
Effect of LE-VPA on the gait parameters after KA. **(A)** Diagram of gait analysis. The Catwalk gait analysis demonstrated that auxiliary running could improve the gait posture as reflected by the increased print area **(B)**, swing speed **(C)**, and body speed **(D)** compared with the model group. **(E–H)** Displayed the footprints picture and the marching patterns of the groups. Data were expressed as mean ± SEM. ns > 0.05, ***P < 0.001; ^#^*P* < 0.05, ^###^*P* < 0.001, comparison of the model group with each treated group (two-way ANOVA).

Motor skill deficits are nearly universal to all neurodevelopmental disorders, and epilepsy is no exception (38). To assess these deficits, mice were exposed to Catwalk analysis. After habituation to the new surroundings, the animals had to perform a minimum of three non-stop runs that qualified for Catwalk XT® analysis. The same calibration parameters were used for each experimental group. To improve data quality, automated footprint recognition was manually reviewed. Catwalk XT® software separately analyzed a combination of 24 dynamic and static parameters for each paw. In addition, the print positions of the left and right paws, as well as the base of the support of the front and hind paws, were analyzed (Figure 4A). In the Catwalk gait analysis, print area and mean intensity indicated the paw force, which may reflect the weight-bearing capability of the limbs; swing speed and body speed indicated the thrust force of the limbs, where shorter swing speed or body speed indicated that the thrust force of the limbs was reduced (Figures 4E–H). Compared with the model group, only the footprint area was significantly increased in the drug group (Figure 4B, *P* < 0.001, *n* = 6). On the contrary, all parameters, print area (*P* < 0.05, *n* = 6), swing speed (Figure 4C, *P* < 0.001, *n* = 6), and body speed (Figure 4D, *P* < 0.01, *n* = 6), were significantly increased in the runner group. Mice in the combination group showed improvement in all three parameters (*P* < 0.001, *n* = 6). In addition, compared with the drug group, the combination group showed a more significant effect on swing speed (*P* < 0.001) and body speed (*P* < 0.001). Therefore, compared with VPA treatment, LE-VPA can significantly improve movement disorders such as discoordination of limbs and slow movement caused by epilepsy.

### Inhibitory effects of LE-VPA on LFP responses

**Figure 5:**
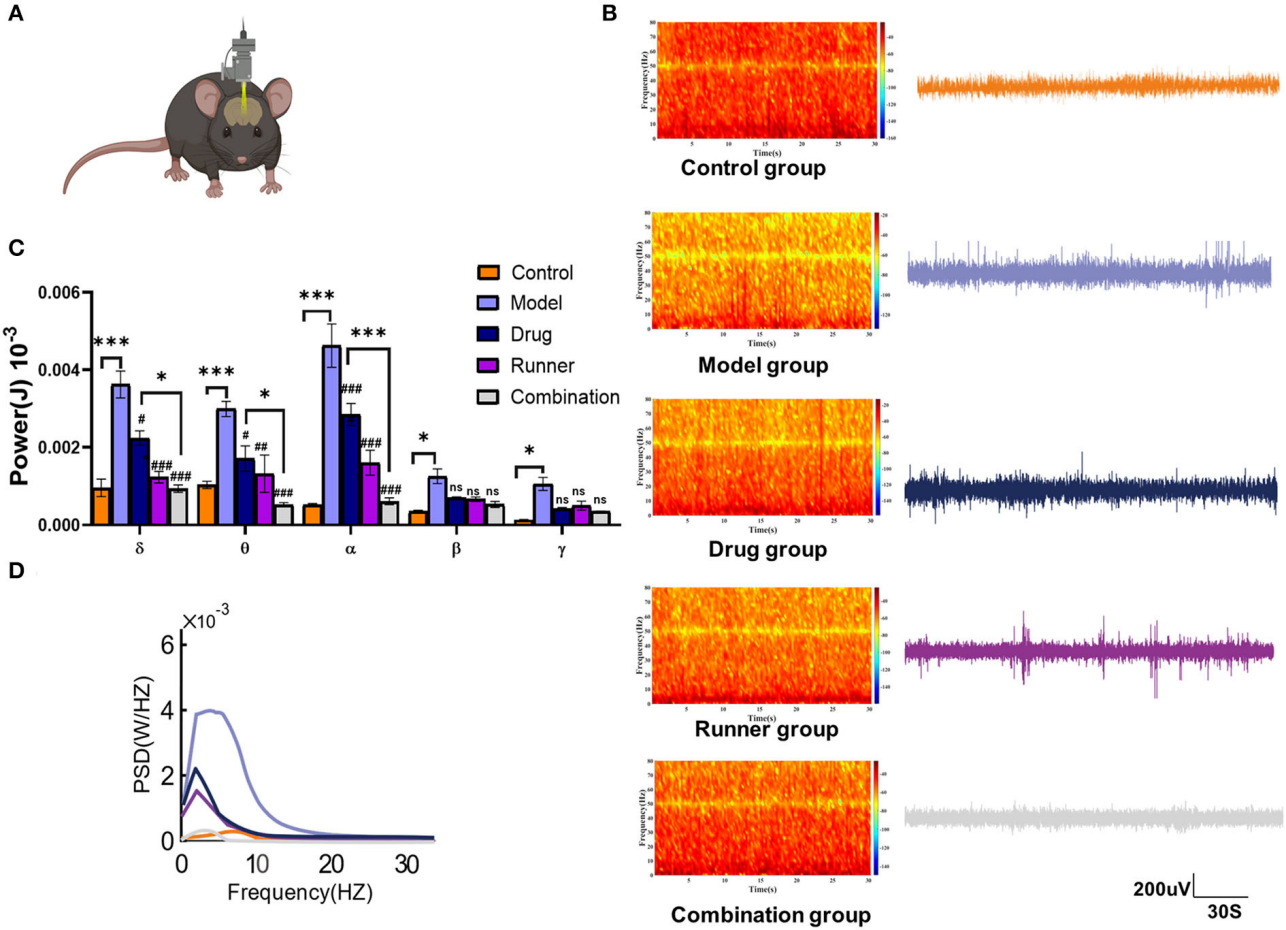
LFP spectral characteristics and PSD in each group. **(A)** Diagram of local field potential recording in mice. **(B)** LFP signals and spectral heat maps from representative seizure mice treated with control (orange), model (lavender), drug (blue), runner (amaranth), and combination (gray) groups were displayed, respectively. **(C)** Spectral plots and cumulative distribution curves from model mice with no treatment, VPA, treadmill, and combination treatment were shown, respectively. **(D)** Spectral analysis on PSD values of δ, θ, α, β, and γ rhythm in each group. Data in **(C)** were shown as mean ± SEM. ns > 0.05, *P < 0.05, ***P < 0.001; ^#^*P* < 0.05, ^##^*P* < 0.01, ^###^*P* < 0.001, compared with model group (two-way ANOVA).

Subsequently, to determine whether LE-VPA could reduce the intensity of epileptic seizures, the effects of running on the power spectral density (PSD) of LFP during KA-induced seizures were directly tested (Figure 5A). In the recording, which was not < 30 min, 10 min of data were randomly selected for statistical analysis. The LFP of control mice showed normal rhythms with low-frequency amplitude, and there was no abnormal epileptic rhythm. The electroencephalogram of model mice showed an explosive polyspike rhythm with high frequency and large amplitude, while the LFP frequency and amplitude were decreased in both the drug and runner groups. On the contrary, the LFP frequency and amplitude in the combination group were almost like those in healthy control mice (Figure 5B). The energy intensity of five common rhythms collected by LFP was statistically analyzed, as shown in Figure 5C. The δ rhythms (0.5–4 Hz) are slow rhythm of the sleep state in mice that can convert early long-term potentiation (LTP) to long-lasting LTP (39). The energy intensity of δ- rhythms of KA mice was significantly increased (*P* < 0.001, *n* = 3). Compared with the model group, the energy intensity of δ rhythms of the drug (*P* < 0.05, *n* = 3), runner (*P* < 0.001, *n* = 3), and combination groups (*P* < 0.001, *n* = 3) decreased by 50 and 70%. The θ rhythms (4–7 Hz) are like δ rhythms and occur during sleep. The KA model group exhibited a higher θ- rhythm energy intensity (*P* < 0.001, *n* = 3). Compared with the KA group, the energy intensity of θ rhythms of the drug group (*P* < 0.05, *n* = 3), runner group (*P* < 0.01, *n* = 3), and combination group (*P* < 0.001, *n* = 3) was decreased by 50%. The α rhythms (8–13 Hz) are normal brain rhythms in mice. The α rhythm energy intensity was significantly increased in the model group (*P* < 0.001, *n* = 3). Compared with the model group, the energy intensity of α rhythms of the drug, running, and combination groups (all *P* < 0.001, *n* = 3) was significantly decreased. The θ and α frequency bands are usually collectively referred to as the low-frequency (LF) range (40, 41). LF oscillations have been widely studied in cognitive and emotional fields, including memory consolidation (42), attention and consciousness (43), and depressive mood (44, 45). There is some evidence that LF oscillations are related to the message of self-movement (46) and sequential dependencies during spatial planning (47). The β rhythms (15–30 Hz) are the main rhythms of the brain during excitation, which seem to focus more on associative memory and ability to predict object locations based on environmental cues (48, 49). The energy intensity of the β rhythms was significantly increased in the model group as compared with the control group (*P* < 0.05, *n* = 3), while the other groups showed no significant difference from the model group. The γ rhythms (> 30 Hz) are fast rhythms occurring in the rapid eye movement sleep period, in which oscillations in entorhinal cortex-hippocampus circuits play crucial roles in memory function in a healthy animal's brain (50). The energy intensity of the γ rhythms was significantly increased in the model group (*P* < 0.05, *n* = 3), while the other groups showed no significant difference as compared with the model group. Figure 5D shows that the PSD of the model group was increased, while it was inhibited to a certain extent after drug therapy, running therapy, and combination therapy. In addition, the combination group showed more effective inhibition of abnormal LFP signals than the drug group for the δ (*P* < 0.05), θ (*P* < 0.05), and α rhythms (*P* < 0.001). These results also support our hypothesis that LE-VPA therapy is more potent in suppressing neuroexcitability, especially low-frequency rhythm.

### Suppressive effect of LE-VPA on neuroinflammatory response induced by epilepsy

**Figure 6:**
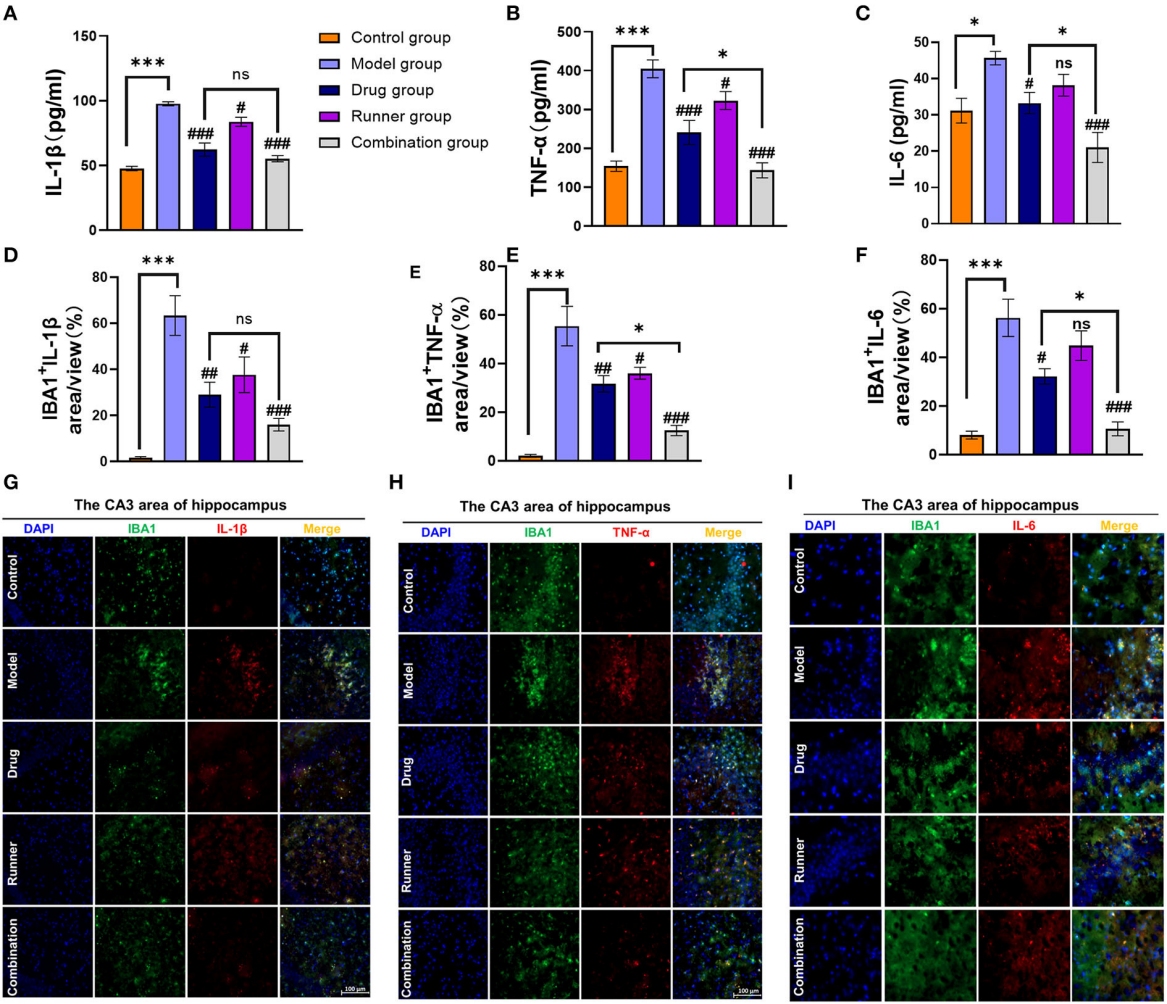
LE-VPA blocked the activation of microglial cells and the release of inflammatory factors. **(A–C)** ELISA analysis of secreted TNF-α, IL-1β, and IL-6 levels in control and four different treatment groups with epilepsy. **(D–F)** Quantitative analysis of percentage of IBA1^+^ IL-1β-, IBA1^+^ TNF-α-, and IBA1^+^ IL-6-positive area. **(G–I)** Immunofluorescent staining of IBA1 and IL-1β, IBA1^+^ TNF-α, and IBA1^+^ IL-6 in the hippocampal CA3 area (scale bar, 100 μm). The data were expressed as the mean ± SEM. ns > 0.05, *P < 0.05, ***P < 0.001; ^#^*P* < 0.05, ^##^*P* < 0.01, ^###^*P* < 0.001, compared with model group (two-way ANOVA).

In mammals, epilepsy can be caused by a large number of inflammatory factors that activate microglial cells (15). We used ELISA (Figures 6A–C) and immunofluorescence (Figures 6D–G), to verify the expression levels of the inflammatory cytokines IL-1β, TNF-α, and IL-6 in the hippocampus of mice. High levels of IL-1β (*P* < 0.001, *n* = 6), TNF-α (*P* < 0.001, *n* = 6), and IL-6 (*P* < 0.05, *n* = 6) were expressed in the hippocampal tissues of model mice, while running reduced the expression levels of IL-1β (*P* < 0.05, *n* = 6) and TNF-α (*P* < 0.05, *n* = 6). Drug treatment alone was effective in reversing the hyperinflammatory state of the hippocampus (IL-1β and TNF-α: *P* < 0.001, IL-6: *P* < 0.05, *n* = 6), but the advantage of the combination group was more prominent (all *P* < 0.01, *n* = 6) and significantly different from that of the drug group in TNF-α and IL-6 expression (both *P* < 0.05, *n* = 6). To verify this conclusion, we performed immunofluorescence staining of IBA1 (M1 microglial marker), IL-1β, TNF-α, and IL-6 in the CA3 region (51) and found that microglial cells were activated and IL-1β, TNF-α, and IL-6 levels were high in model mice (all *P* < 0.001, *n* = 6). We determined the average percentage of positive areas of IBA1^+^ IL-1β, IBA1^+^ TNF-α, or IBA1^+^ IL-6 in each group. Compared to the model group, the levels of all three inflammatory factors in the hippocampus decreased after treatment with VPA alone (IL-1β and TNF-α: *P* < 0.01, IL-6: *P* < 0.05, *n* = 6). In the combination group, the mean positive area related to IL-1β, TNF-α, and IL-6 was decreased by 60% (*P* < 0.001, *n* = 6), 50% (*P* < 0.001, *n* = 6), and 50% (*P* < 0.001, *n* = 6), respectively. Compared with the drug group, LE-VPA has more obvious inhibitory effect on the TNF-α (*P* < 0.05) and IL-6 (*P* < 0.05).

### LE-VPA inhibited NF-κB activation in the hippocampus

**Figure 7:**
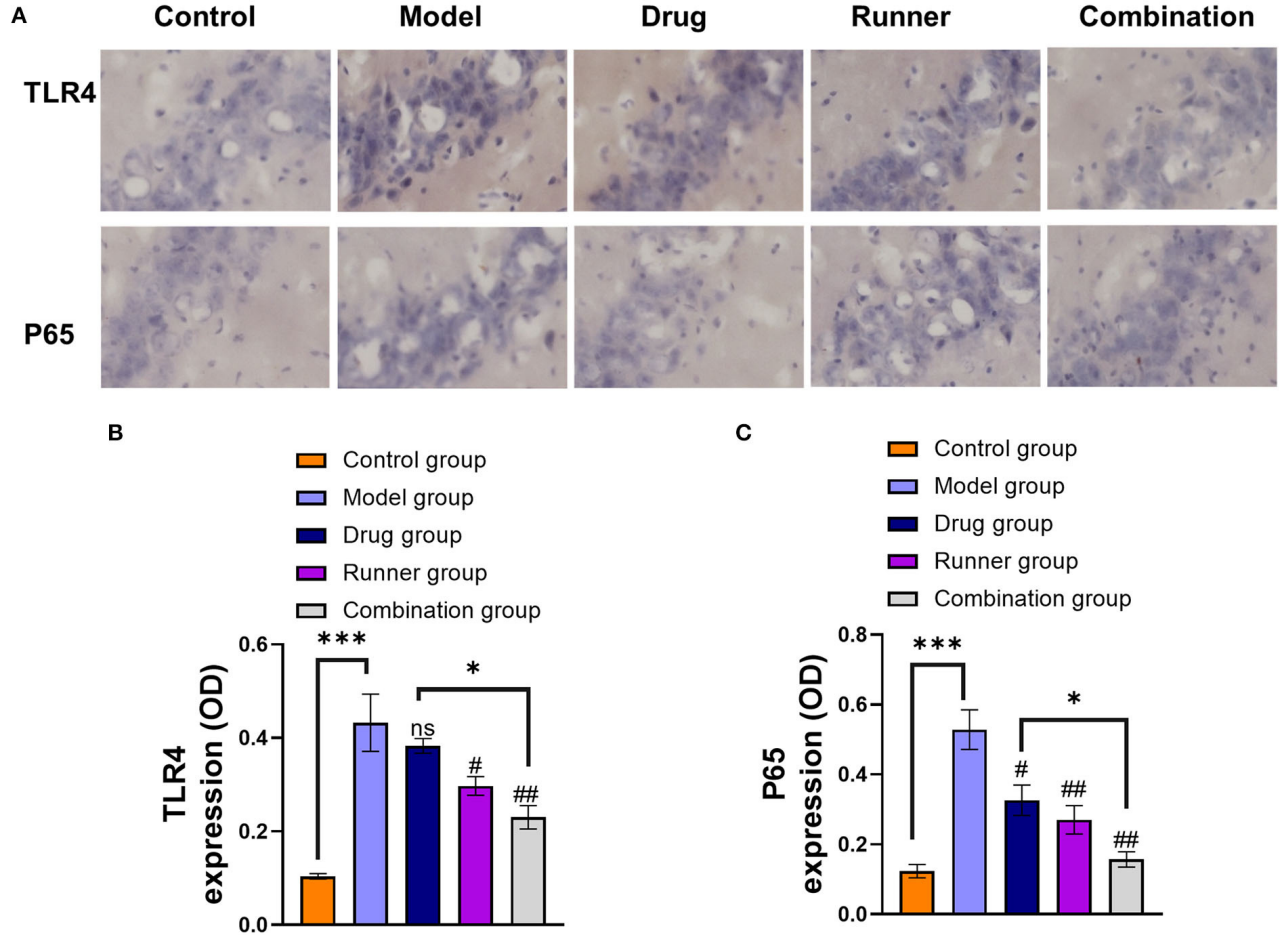
Effect of LE-VPA on TLR4/ NF-kB activation in the hippocampus. **(A)** Five brain slices were processed for TLR4 and NF-κB p65 immunohistochemical staining. Experiments were performed three times, and the staining of the cells under a 20x microscope was shown. **(B,C)** Comparison of the IODs of TLR4 and p65 using immunohistochemical staining in the hippocampus from each group. The data were expressed as the mean ± SEM. ns > 0.05, *P < 0.05, ***P < 0.001; ^#^*P* < 0.05, ^##^*P* < 0.01, compared with model group (two-way ANOVA).

In the attempt to define the underlying molecular mechanisms by which LE-VPA suppressed microglial inflammatory response, we monitored expression levels of TLR4 and the activation of the transcription factor NF-κB, which occurs in response of proinflammatory stimuli and results in increased expression of many cytokines and chemokines (52). To this end, we quantified the expression level of NF-κB p65 subunit as an indicator of NF-κB activation (Figures 7A–C). TLR4 (*P* < 0.001, *n* = 6) and p65 (*P* < 0.001, *n* = 6) are abundant in the hippocampus of KA mice, and the levels of these two factors decreased in the runner group (TLR4: *P* < 0.05, P65: *P* < 0.01, *n* = 6) and combination group (both *P* < 0.01, *n* = 6) after exercise, but drug group only reversed the expression of p65 (*P* < 0.05, *n* = 6) and has no effect on TLR4 (*P* > 0.05, *n* = 6). Thus, the combination treatment significantly reduced the expression of TLR4 and inhibited NF-κB activation compared with the drug alone treatment (*P* < 0.05), suggesting that inhibition of NF-κB activation may contribute to the anti-inflammatory effect of the microglial cells.

## Discussion

**Figure 8:**
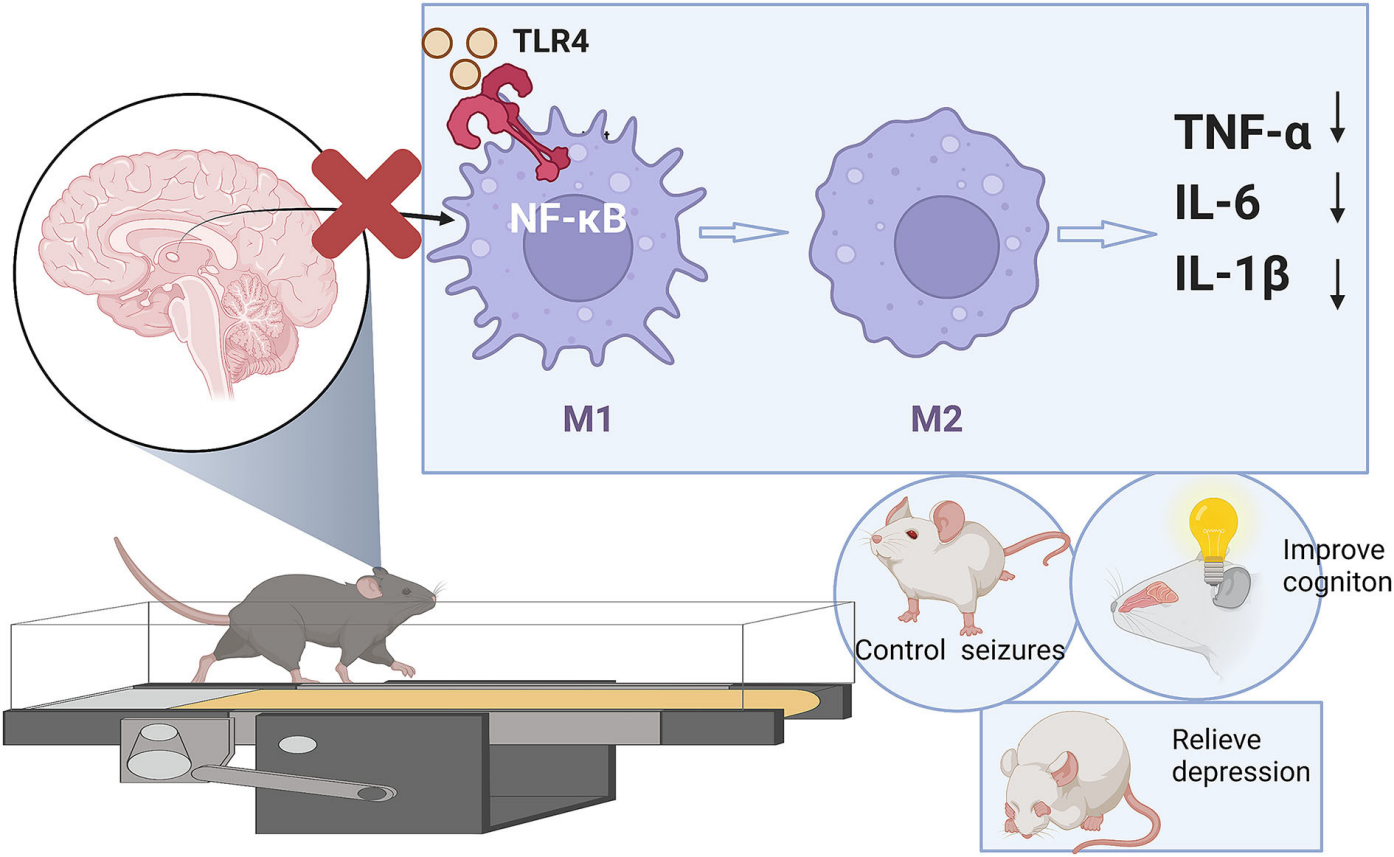
Summary of the article.

First, our results of animal behavioral experiments and LFP show that compared with conventional drug treatment, LE-VPA can better control mild seizures and ease epilepsy-associated co-morbidities, such as cognitive dysfunction, depression, anxiety-like behaviors, and movement disorders (Figures 2–4). In addition, we found that LE-VPA had a larger significant effect on the abnormal LFP signal at low-frequency waves (Figure 5). This result is consistent with the literature, since LFP is used to understand the relationship between brain oscillations within different frequency ranges and cognitive and motor processes (53). Next, we explored the mechanism of action of the LE-VPA treatment of seizures and epilepsy-associated co-morbidities. We performed immunohistochemistry to examine the activation of TLR4/ NF-κB and the downstreaming IL-1β, TNF-α, and IL-6 signaling in the hippocampus. LE-VPA reduced TLR4 expression, inhibiting NF-κB signaling and, in turn, inhibiting the increase in expression levels of IL-1β, TNF-α, and IL-6. Therefore, this novel therapeutic approach was more effective in attenuating seizures and associated co-morbidities (Figure 8).

The pathogenesis of epilepsy is complex and has not been fully elucidated; consequently, there is a limited clinical cure rate. Recent studies have suggested that a variety of signaling pathways may be associated with epilepsy (54). These pathways include the Phosphatidylinositol-3-kinase (PI3K) / protein kinase B (Akt) / mammalian target of rapamycin (mTOR) signaling pathway that regulates the autophagy process in neurodegenerative diseases (55), the Wnt/β-catenin signaling pathway (56), mitogen-activated protein kinase (MAPK) signaling pathway (57) and the TLR4/ NF-κB pathway (58) that is associated with neurogenesis and neuronal death. Different inflammatory factors are closely related to these signaling pathways. Studies have shown that an increase in the BBB permeability is directly associated with seizure generation and severity (18, 59). Activated macrophages produce IL-1, IL-6, and TNF-α; these cytokines can alter the permeability of the blood–brain barrier (BBB) and allow peripheral proinflammatory factors to enter the circumventricular brain regions and cause seizures (60), followed by the activation of NF-κB pathway. IL-1β then triggers the breakdown of the BBB and activates glial cells (61). NF-κB then recruits other proteins (coactivators and RNA polymerase), which finally lead to the expression of proinflammatory cytokines (e.g., IL-1, IL-2, IL-6, and TNF-α) that exacerbates and perpetuates the seizures (62). In addition, TNF-α can control the glutamate receptor transport *via* TNF receptor 1 and the TNF receptor 2, and these receptors promote neuron firing by increasing glutamate levels in the synaptic cleft; therefore, TNF-α plays a role in epilepsy (63). During acute and chronic seizures, IL-1β is highly expressed and its binding to IL-1β receptors (IL-1R1) activates NF-κB in target cells amplifying the inflammatory response (64). Previous studies have shown that following seizures, the IL-6 receptor mRNA is upregulated only in the hippocampus (65, 66). However, in recent years, there have been conflicting reports on the role of IL-6 in seizures. Although IL-6 is necessary for the nervous system normal development (67), high levels of IL-6 in the brain can lead to neurotoxic and proconvulsive effects (68, 69). The inhibition of the histone deacetylase (HDAC) and the modulation of brain-derived neurotrophic factor (BDNF) play a key role in regulating the main pathways that modulate the VPA epigenetic effects (70). Therefore, VPA may inhibit PI3K/Akt/ Mouse double minute 2 (MDM2) signaling pathway (71), inhibit NF-κB, p65-dependent transcriptional activation (71), and promote the tropomyosin receptor kinase B (TrkB)/BDNF signaling pathway (70) that reduces neuroinflammation and regulate synaptic plasticity.

In recent years, the relationship between VPA and cognitive function has been controversial (72–74). Our results of the water maze and the Y maze align with the evidence that shows VPA-induced cognition improvement in KA mice. There was no significant difference between the VPA-treated mice and the control group in total distance traveled in the Y maze (Figure 3G); this result could be explained by the diminished motor state of the mice resulting from the continuous intraperitoneal drug injection for 4 weeks. The gait analysis showed that VPA increased the print area during walking, but not the swing speed or body speed (Figure 4). This result suggests VPA might improve walking stability in patients with epilepsy without changing their physical flexibility. In addition, the immunohistochemistry showed that VPA did not reduce the expression levels of TLR4 in the hippocampus but significantly inhibited NF-κB p65-dependent transcriptional activation (Figure 6). We speculate that VPA may inhibit NF-κB activation through a pathway other than TLR4.

There is vast evidence for the beneficial effects of physical exercise on epilepsy in animals models and at the clinical level (75). For instance, it improves cognitive function and alleviates depression and anxiety in patients with epilepsy, which is consistent with our experimental results. The exact mechanism has not been elucidated. Nevertheless, researchers have adopted different strategies to explain the phenomenon. First, in chronic epilepsy, aerobic exercise improves regional cerebral glucose metabolism and an increase in adenosine content in the brain gray matter associated with motor, sensory, and autonomic functions. Thus, aerobic exercise has an anticonvulsant effect by affecting brain metabolism (76). Second, physical exercise exerted positive effects on hippocampal synaptic plasticity, including increasing hippocampal neurogenesis and restoring the LTP-induced damage in epileptic rats (77). Particularly, physical exercise alters BDNF/TrkB (78) and Akt/ mTOR signaling pathways (79). Finally, physical exercise can improve the hyperinflammatory state in the hippocampus (80). It has been shown that swimming exercise stimulates IGF1/PI3K/Akt and AMPK/SIRT1/PGC1α survival signals to suppress inflammation (81).

Most studies suggest that physical exercise can boost serotonin (82), norepinephrine (83), dopamine synthesis and release (84), increase BDNF, and reduce the activity of the hypothalamus–pituitary–adrenal (85). Consequently, there is a reduction in epilepsy co-morbidities. In addition, studies have shown that moderate physical exercise could reduce the levels of IL-1β and TNF-α in the hippocampus or serum (86, 87) in interventions for brain disorders, although the effect on IL-6 is subject to specific discussion. To illustrate, physical exercise can reduce IL-6 levels in the hippocampus and cerebellum (88, 89). By contrast, during physical exercise, skeletal muscle produces large amounts of IL-6 to induce hepatic glucose export and induce lipolysis (90).

Altogether, our experiments showed that VPA plays a leading role in improving the frequency of severe seizures; however, physical exercise alone has little effect as a treatment (Figures 2C,D). Moreover, the effects of physical exercise alone on working memory tests (Figure 3E) and hippocampal IL-6 expression levels (Figures 6C,F) were also limited. Considering previous studies, we speculate that the physical exercise intensity may not be the optimal intensity for KA mice. Therefore, the next step is to determine the optimal intensity for the KA mice. To this end, we will divide the physical exercise intensity by gradient. Here, we showed that low-intensity physical exercise may be inhibiting the TLR4/ NF-κB pathway. Nonetheless, the mechanism in which the physical exercise-generated force is affecting the TLR4 expression in the brain needs to be further explored. For instance, physical exercise activates the mechanical sensor Piezo1 (91) which leads to enhanced expression of the bone-derived growth factor osteocalcin (OCN) (92). Subsequently, OCN can regulate cognition through G protein-coupled receptors (GPR) (93, 94). Furthermore, it has been shown that activation of GPR30 on microglial cells can reduce ischemic injury by inhibiting TLR4-mediated microglial inflammation. We need more evidence on whether microglial cells have receptors for OCN, thus, possibly playing a role in seizures and its co-morbidities by regulating the TLR4-mediated signaling pathway. These results suggest the LE-VPA treatment is a promising novel therapeutic avenue with translational application.

## Data availability statement

The original contributions presented in the study are included in the article/supplementary material, further inquiries can be directed to the corresponding authors.

## Ethics statement

The animal study was reviewed and approved by Shanghai University of Traditional Chinese Medicine.

## Author contributions

YJi, JT, and YJia designed the research. YJia and LT performed the data analysis work. YJia, LT, YY, XC, and LZ conducted experiments. YJia wrote and revised the manuscript. LT and DQ drew the figures. YZ was responsible for the statistical analyses. All authors read and approved the final manuscript.

## Funding

This work was supported by grants from National Key Research and Development Program (No. 2020YFA0803800), National Natural Science Foundation of China (Nos. 82274344, 82074162, and 81903995), Science, Technology Innovation Project of Putuo District Health System (No. ptkwws202107), Research Project (2020336B), and the One Hundred Talents Project of Putuo Hospital, Shanghai University of Traditional Chinese Medicine (No. 2022LH002).

## Conflict of interest

The authors declare that the research was conducted in the absence of any commercial or financial relationships that could be construed as a potential conflict of interest.

## Publisher's note

All claims expressed in this article are solely those of the authors and do not necessarily represent those of their affiliated organizations, or those of the publisher, the editors and the reviewers. Any product that may be evaluated in this article, or claim that may be made by its manufacturer, is not guaranteed or endorsed by the publisher.
